# Estimation of child vaccination coverage at state and national levels in India

**DOI:** 10.2471/BLT.15.167593

**Published:** 2016-06-21

**Authors:** Pankaj Bhatnagar, Satish Gupta, Rakesh Kumar, Pradeep Haldar, Raman Sethi, Sunil Bahl

**Affiliations:** a World Health Organization Country Office for India, Nirman Bhawan, Maulana Azad Road, New Delhi, India.; b United Nations Children’s Fund Country Office for India, New Delhi, India.; c Ministry of Health and Family Welfare, Government of India, New Delhi, India.; d World Health Organization Regional Office for South-East Asia, New Delhi, India.

## Abstract

**Objective:**

To review the data, for 1999–2013, on state-level child vaccination coverage in India and provide estimates of coverage at state and national levels.

**Methods:**

We collated data from administrative reports, population-based surveys and other sources and used them to produce annual estimates of vaccination coverage. We investigated bacille Calmette–Guérin vaccine, the first and third doses of vaccine against diphtheria, tetanus and pertussis, the third dose of oral polio vaccine and the first dose of vaccine against measles. We obtained relevant data covering the period 1999–2013 for each of 16 states and territories and the period 2001–2013 for the state of Jharkhand – which was only created in 2000. We aggregated the resultant state-level estimates, using a population-weighted approach, to give national values.

**Findings:**

For each of the vaccinations we investigated, about half of the 253 estimates of annual coverage at state level that we produced were based on survey results. The rest were based on interpolation between – or extrapolation from – so-called anchor points or, more rarely, on administrative data. Our national estimates indicated that, for each of the vaccines we investigated, coverage gradually increased between 1999 and 2010 but then levelled off.

**Conclusion:**

The delivery of routine vaccination services to Indian children appears to have improved between 1999 and 2013. There remains considerable scope to improve the recording and reporting of childhood vaccination coverage in India and regular systematic reviews of the coverage data are recommended.

## Résumé

**Objectif:**

Examiner, pour la période 1999-2013, les données relatives à la couverture vaccinale des enfants au niveau étatique en Inde et fournir des estimations de la couverture vaccinale aux niveaux étatique et national.

**Méthodes:**

Nous avons rassemblé des données provenant, entre autres sources, de rapports administratifs et d'enquêtes auprès de la population pour obtenir des estimations annuelles de la couverture vaccinale. Notre étude a porté sur le vaccin bilié de Calmette et Guérin, la première et la troisième dose du vaccin contre la diphtérie, le tétanos et la coqueluche, la troisième dose du vaccin antipoliomyélitique oral et la première dose du vaccin contre la rougeole. Nous avons obtenu des données pertinentes couvrant la période 1999-2013 pour 16 États et territoires et la période 2001-2013 pour l'État du Jharkhand, qui a vu le jour en 2000 seulement. Nous avons ensuite regroupé les estimations au niveau étatique qui en découlaient à l'aide d'une approche pondérée selon la population afin d'obtenir des valeurs nationales.

**Résultats:**

Pour chaque type de vaccination étudié, environ la moitié des 253 estimations de la couverture annuelle au niveau étatique que nous avons établies était basée sur des résultats d'enquêtes. Les autres reposaient sur une interpolation entre les points d'ancrage – ou une extrapolation à partir des points d'ancrage – ou, plus rarement, sur des données administratives. Nos estimations nationales faisaient état, pour chacun des vaccins étudiés, d'une augmentation progressive de la couverture vaccinale entre 1999 et 2010, puis d'une stagnation.

**Conclusion:**

La prestation de services de vaccination systématique à l'intention des enfants indiens semble s'être améliorée entre 1999 et 2013. Il reste néanmoins encore beaucoup à faire pour favoriser l'enregistrement et l'établissement de rapports sur la couverture vaccinale des enfants en Inde et il est recommandé de réaliser des études systématiques régulières sur les données relatives à la couverture.

## Resumen

**Objetivo:**

Revisar los datos de los años 1999 a 2013 sobre la cobertura de vacunación infantil a nivel estatal en la India y ofrecer estimaciones de cobertura a nivel estatal y nacional.

**Métodos:**

Se recopilaron datos de informes administrativos, encuestas basadas en la población y otras fuentes y se utilizaron para producir estimaciones anuales de la cobertura de vacunación. Se investigaron la vacuna del bacilo Calmette–Guérin (BCG), la primera y tercera dosis de la vacuna contra la difteria, el tétanos y la tos ferina, la tercera dosis de la vacuna antipoliomielítica oral y la primera dosis de la vacuna contra el sarampión. Se obtuvieron datos relevantes del periodo comprendido entre 1999 y 2013 para cada uno de los 16 estados y territorios y del periodo comprendido entre 2001 y 2013 para el estado de Jharkhand, que se creó en el año 2000. Se añadieron las previsiones resultantes a nivel estatal, utilizando un enfoque de ponderación poblacional, para obtener los valores nacionales.

**Resultados:**

Para cada una de las vacunas investigadas, alrededor de la mitad de las 253 estimaciones de cobertura anual a nivel estatal que se produjeron se basaron en los resultados de encuestas. El resto se basaron en la interpolación entre, o extrapolación de, los conocidos puntos de anclaje o, con menos frecuencia, datos administrativos. Las estimaciones nacionales indicaron que la cobertura para cada una de las vacunas investigadas aumentó de forma gradual entre los años 1999 y 2010, pero luego se mantuvo sin cambios.

**Conclusión:**

La prestación de servicios de vacunación rutinarios a niños indios parece haber mejorado entre los años 1999 y 2013. Aún queda mucho margen para mejorar el registro y la presentación de informes sobre la cobertura de vacunación infantil en la India, y se recomienda realizar revisiones sistemáticas de forma regular de la información sobre la cobertura.

## ملخص

**الغرض:**

مراجعة البيانات، فيما يتعلق بالفترة من عام 1999 حتى عام 2013، بشأن تغطية خدمات تطعيم الأطفال على مستوى الولاية في الهند وتقديم تقييمات لتغطية تلك الخدمات على مستوى الولاية والمستوى الوطني.

**الطريقة:**

جمعنا البيانات من التقارير المستمدة من السجلات والمسوح المستندة إلى عينات سكانية وغير ذلك من المصادر واستخدمناها للوصول إلى تقييمات سنوية لتغطية خدمات التطعيم. وأجرينا استقصاءًا للقاح عصيات كالميت – غيران، والجرعتين الأولى والثالثة من اللقاح المضاد للدفتريا والتيتانوس والسعال الديكي، والجرعة الثالثة من اللقاح الفموي لشلل الأطفال، والجرعة الأولى من اللقاح المضاد للحصبة. وحصلنا على البيانات ذات الصلة والتي تشمل الفترة من عام 1999 حتى عام 2013 فيما يتعلق بـ 16 ولاية وإقليم كل على حدة، والفترة من عام 2001 حتى عام 2013 في ولاية "جهارخاند" - التي لم يتم إنشاؤها إلا في عام 2000 فقط. وجمّعنا التقييمات التي أثمرت عنها تلك البيانات على مستوى الولاية، باستخدام أسلوب الترجيح وفقًا لعينات السكان لتقديم قيم على المستوى الوطني.

**النتائج:**

أظهر الاستقصاء الذي أجريناه لكل نوع من أنواع اللقاحات أن حوالي نصف التقييمات التي توصلنا إليها والبالغ عددها 253 تقييم بشأن التغطية السنوية على مستوى الولاية اعتمدت على نتائج المسوح. واعتمدت التقييمات الباقية على الاستكمال الداخلي بين – أو الاستكمال الخارجي بناءً على - ما يُطلق عليه اسم النقاط المعروفة سلفًا أو، في حالات أكثر ندرة، بيانات مستمدة من السجلات. وأشارت التقديرات التي توصلنا إليها على المستوى الوطني إلى الزيادة التدريجية في معدل التغطية المتعلقة بكل نوع من أنواع اللقاحات الخاضعة للاستقصاء في الفترة بين 1999 و2010، ولكنه شهد استقرارًا منذ ذلك الحين.

**الاستنتاج:**

يبدو أن تقديم خدمات التطعيم المعتادة للأطفال الهنود قد تحسن في الفترة بين عامي 1999 و2013، ولكن لا تزال الفرصة سانحة بدرجة كبيرة لتحسين تسجيل تغطية خدمات تطعيم الأطفال والإبلاغ بها في الهند، كما نوصي بإجراء مراجعات منهجية بانتظام للبيانات المتعلقة بالتغطية.

## 摘要

**目的:**

回顾 1999 年至 2013 年间印度邦级儿童疫苗接种覆盖率的相关数据并预测邦级和国家级疫苗接种覆盖率。

**方法:**

我们收集了来自管理报告、基于人群的调查以及其它来源的数据，并应用这些数据得出疫苗接种覆盖率的年度预测。我们调查了卡介苗、第一剂和第三剂百白破疫苗、第三剂口服小儿麻痹疫苗以及第一剂麻疹疫苗，收集了 1999 年至 2013 年间 16 个印度邦及联邦属地的相关数据和 2001 年至 2013 年间贾坎德邦的相关数据（于 2000 年创建)，并使用人口加权方法整合邦级预测结果，从而得出国家级数据。

**结果:**

对于我们调查的所有疫苗接种情况，在我们建立的 253 项邦级年度疫苗接种覆盖率预测中，大约有一半数据是基于我们的调查结果。其余数据则是基于所谓的锚点数据，或者（更少见的）管理型数据通过内插法——或者外推法得出。我们的国家级疫苗接种覆盖率的预测结果显示，从 1999 年至 2010 年间，我们调查的每种疫苗接种覆盖率均逐渐增加，但之后则保持在平稳状态。

**结论:**

在 1999 年至 2013 年间，印度儿童的例行性疫苗接种服务提供情况似乎有所改善。但印度儿童疫苗接种覆盖率的记录和报告方面仍有大幅提升空间，建议对覆盖率数据进行定期系统性回顾。

## Резюме

**Цель:**

Изучить данные за период 1999–2013 гг., относящиеся к охвату детской вакцинацией на уровне штатов в Индии, и рассчитать показатели охвата на уровне штатов и национальном уровне.

**Методы:**

Данные из административных сводок, популяционных опросов и других источников были систематизированы и использованы для определения годовых показателей охвата вакцинацией. Объектами исследования являлись вакцина БЦЖ, первая и третья доза вакцины против дифтерии, коклюша и столбняка, третья доза пероральной вакцины против полиомиелита и первая доза вакцины против кори. Релевантные данные за период 1999–2013 гг. были получены для каждого из 16 штатов и территорий и за период 2001–2013 гг. для штата Джаркханд, который был создан в 2000 г. Результирующие показатели на уровне штатов были объединены с пересчетом на численность населения, что позволило вывести значения для национального уровня.

**Результаты:**

Для каждой исследованной вакцины приблизительно половина из 253 полученных авторами показателей ежегодного охвата на уровне штатов основывалась на результатах опросов. Остальные показатели были получены на основании интерполяции между так называемыми опорными точками или экстраполяции из них или (реже) на основании административных данных. Судя по полученным национальным показателям, охват каждой исследованной вакцинацией постепенно увеличивался между 1999 и 2010 годами, но затем стабилизировался.

**Вывод:**

Было выявлено, что в период между 1999 и 2013 годом происходило увеличение масштабов оказания услуг плановой вакцинации индийских детей. Процесс регистрации и сообщения данных по охвату детской вакцинацией в Индии по-прежнему может быть улучшен во многих аспектах. Авторы рекомендуют проводить регулярные систематические обзоры данных по охвату.

## Introduction

The landscape of routine child immunization in India is changing rapidly.^1^^–^^3^ The national government declared 2012–2013 to be a period of intensification in child immunization, with a focus on remote and often inaccessible rural areas, urban slums and migrant and mobile communities.^4^ Subsequently, in December 2014, India’s Ministry of Health and Family Welfare launched Mission Indradhanush. The aims of this initiative were to vaccinate at least 90% of pregnant women against tetanus and ensure that all children are fully vaccinated against seven vaccine-preventable diseases before they reach an age of two years.^5^^,^^6^ General improvements in the delivery of routine immunization services were also critical in the successful efforts to interrupt polio transmission in India and remain a key component in attempts to eliminate measles from the country by 2020.^7^

The monitoring of trends in vaccine coverage is complicated by the multiple sources of relevant data and the varied quality of those sources. Although there are data available on administrative coverage – i.e. data on the immunization services delivered by health providers – potentially useful information on vaccination coverage is also collected in coverage evaluation surveys, process and community monitoring, surveillance on vaccine-preventable diseases, integrated disease surveillance and the management of the cold chains used in the storage and transport of vaccines.

In India, as elsewhere, the accuracy of estimates of administrative coverage depends on the accurate recording of the numbers of administered doses, accurate information on the size of the target population, regular and robust reporting by the health workers who administer the vaccines and the prompt and accurate transfer of the relevant data through all of the levels between the health subcentres and the national government. The numerators and/or denominators needed to calculate percentage coverage values are often only available as rough estimates.

As nationally representative surveys of vaccination coverage are expensive and time-consuming, they tend to be infrequent and poor at providing rapid information on the trends in a system’s or programme’s performance. In India, the last survey of this type was conducted in 2008. Since then, administrative coverage data have served as the primary vehicle for the annual monitoring of vaccination coverage and programme performance at national level. To provide timely feedback, house-to-house rapid monitoring and modified session monitoring have also been implemented – mainly in support of polio-related efforts to strengthen community-based routine immunization. The value of data collected by rapid monitoring is, however, often reduced by selection bias – e.g. as a result of the monitoring being confined to communities that are considered to be at relatively high risk of vaccine-preventable disease – and the challenges posed by determining the size of the target population accurately.

In an attempt to improve our knowledge of recent trends in child vaccination coverage in India, we recently collected relevant data from multiple information sources and used them to derive estimates of the annual levels of such coverage, at both state and national level, for the years 1999–2013.

## Methods

As part of a workshop held on 30 April–1 May 2015, with state representatives, we – i.e. the members of a review team that included staff from India’s national immunization programme and their counterparts from the India-based offices of the World Health Organization (WHO) and United Nations Children’s Fund (UNICEF) and WHO’s Regional Office for South-East Asia – reviewed data obtained after 1998 on child vaccination coverage. We investigated data – on bacille Calmette–Guérin vaccine, the first and third doses of diphtheria-tetanus-pertussis (DTP1 and DTP3) vaccine, the third dose of oral polio vaccine and the first dose of vaccine against measles – from state-specific administrative reports, coverage surveys (Table 1) and rapid monitoring. We confined our investigation to data from the 17 states that together accounted for over 95% (24.45 million/25.59 million) of the 2013–2014 birth cohort: Andhra Pradesh, Assam, Bihar, Chhattisgarh, Gujarat, Haryana, Jharkhand, Karnataka, Kerala, Madhya Pradesh, Maharashtra, Odisha, Punjab, Rajasthan, Tamil Nadu, Uttar Pradesh and West Bengal.

**Table 1:** Survey-based assessments of vaccine coverage, 17 states, India, 2002–2013

Characteristic	Survey^a^									
	DLHS2	CES	NFHS3	CES	DLHS3	CES	AHS1	AHS2	AHS3	DLHS4
**Survey year**	2002–2004	2005	2005–2006	2006	2007–2008	2009	2010–2011	2011–2012	2012–2013	2012–2013
**Birth cohort**	2002	2004	2005	2005	2007	2008	2009	2010	2011	2012
**States covered**^b^										
Andhra Pradesh	+	+	+	+	+	+	–	–	–	+
Assam	+	+	+	+	+	+	+	+	+	–
Bihar	+	+	+	+	+	+	+	+	+	–
Chhattisgarh	+	+	+	+	+	+	+	+	+	–
Gujarat	+	+	+	+	+	+	–	–	–	–
Haryana	+	+	+	+	+	+	–	–	–	+
Jharkhand	+	+	+	+	+	+	+	+	+	–
Karnataka	+	+	+	+	+	+	–	–	–	+
Kerala	+	+	+	+	+	+	–	–	–	+
Madhya Pradesh	+	+	+	+	+	+	+	+	+	
Maharashtra	+	+	+	+	+	+	–	–	–	+
Odisha	+	+	+	+	+	+	+	+	+	–
Punjab	+	+	+	+	+	+	–	–	–	+
Rajasthan	+	+	+	+	+	+	+	+	+	–
Tamil Nadu	+	+	+	+	+	+	–	–	–	+
Uttar Pradesh	+	+	+	+	+	+	+	+	+	
West Bengal	+	–	+	+	+	+	–	–	–	+

AHS: Annual Health Survey; CES: Coverage Evaluation Survey; DLHS: District Level Health Survey; NFHS: National Family Health Survey.

^a^ States covered and not covered by a survey are indicated + and – respectively.

^b^ Only the 17 states with the largest birth cohorts for 2013–2014 are shown. Other states and territories were covered by the surveys.

In India, data on administrative coverage are reported for each fiscal year, with each such year running from 1 April in one year to 31 March of the following year. We investigated such data for the period beginning either 1 April 1999 – for 16 of the study states – or 1 April 2001 – for Jharkhand, which was only created in 2000 – and ending 31 March 2014. We used these data and the other relevant data that were available to estimate coverage for each of the corresponding 12-month birth cohorts. To save space, we used the year in which a 12-month birth cohort began to name that cohort – e.g. the birth cohort that began on 1 April 2014 and ended on 31 March 2015 was known as the 2014 birth cohort. To be included in estimations, state-specific survey results had to have been collected in population-based sampling and appear representative at state level.

We investigated vaccination histories recorded, for individual children, on vaccination cards and other home-based records – e.g. the frequency of drop-out between DTP1 and DTP3. When possible, for children aged 12–23 months at the time of survey, we also compared the vaccination history detailed on home-based records with that recalled by the relevant caregivers.

Data were organized, by state, vaccine and year, in an Excel (Microsoft, Redmond, United States of America) database. Temporal trends in coverage in each year and state were displayed graphically, with a different symbol used for each type of information source. The relevant state representatives attending the workshop were asked to clarify and explain the trends and any apparent discrepancies observed in each state.

For the data review and estimation process, we followed the domain-specific and logical inference rules previously used by WHO and UNICEF to estimate coverage levels in 195 countries.^8^^,^^9^ For example, if there were no other relevant data available – or, at least, no other data that indicated a coverage value that differed by more than 10 percentage points from the reported administrative coverage – we took a reported administrative coverage as our estimate of the actual coverage. We also adopted the principles that reported state-level coverage could not exceed 100% and that any observations of large year-to-year decreases or increases in coverage are unlikely to be accurate unless there is some reasonable explanation – e.g. a vaccine stock-out or a strike by health workers. If no such explanation was apparent, we assumed that any data suggesting a year-to-year change in coverage of more than 10% were inaccurate and ignored them.

When, for a given year, state and vaccine coverage data were available from at least two different sources – e.g. from a report of administrative coverage and a survey of coverage – the resultant estimate of coverage was considered to be relatively accurate and categorized as a so-called anchor point. For most of the study states, the first anchor point was established in 2002 – coinciding with India’s second District Level Health Survey. If the data from a survey did not support the corresponding reported administrative coverage – i.e. if it did not indicate a coverage that was within 10 percentage points of the reported administrative coverage – we based our estimate on the survey data.

If missing or ignored data meant that coverage for a state and vaccine could not be estimated directly for a particular year, the coverage for that year was estimated, from the values for the closest anchor points before and after the year, by interpolation. If there were no anchor points after the year, nearest-neighbour extrapolation was used to estimate coverage.

After producing a time series for each state–vaccine combination, we compared estimates across vaccines to ensure internal consistency and tried to resolve any apparent anomalies – e.g. substantial differences in the coverage for DTP3 and the third dose of oral polio vaccine. Whenever data from two or more information sources appeared to conflict, we tried to identify the most accurate source by consideration of the possible biases. We always documented the decisions underlying each estimate.

We aggregated state-level estimates to give national values – assuming that coverage in each of the 18 states and territories not included in our data review was 100%. Using estimated annual birth data from the Central Statistics Office, we computed state-specific weights for each study year by dividing the annual estimated number of births in each state by the corresponding estimated total number of births in India. The weighted national mean coverage for each vaccine and year was then computed by multiplying each state-level coverage by a state-specific population weight and then summing across the 35 states and territories.

In a sensitivity analysis, we assumed that, for each vaccine–year combination, the coverage in each of the 18 unreviewed states was either the mean value for the 17 reviewed states or the lowest coverage value recorded for any of those 17 states.

## Results

**Fig. 1:**
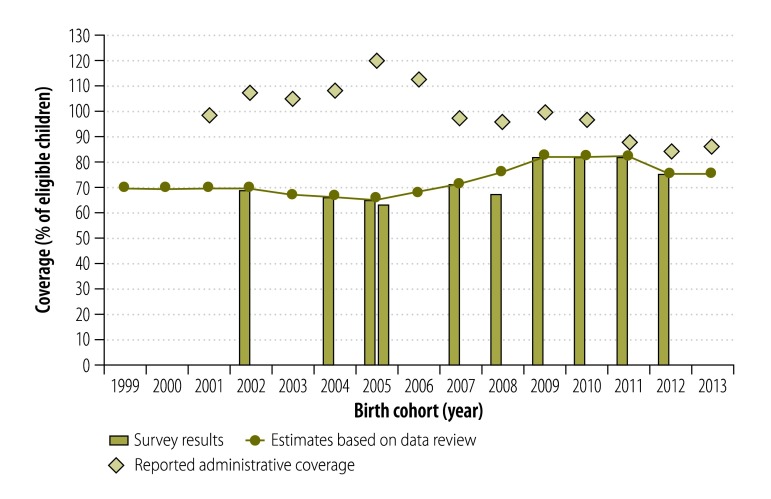
Estimates of the coverage for routine child immunization with the third dose of vaccine against diphtheria, tetanus and pertussis in the state of Chhattisgarh, India, 1999–2013

Notes: The three different sets of estimates that are shown are based on the data review described in this article, reported administrative coverage and surveys for the evaluation of coverage. The absence of a bar for a particular cohort indicates the absence of relevant survey data – and not that a survey indicated zero coverage of that cohort.

The full data on every state-level estimate for each study year and vaccine are available from the corresponding author. As an exemplar of our results, Fig. 1 shows the empirical data and our coverage estimates for one type of vaccination – DTP3 – in one of the study states – Chhattisgarh. For this vaccine–state combination and the 2005 birth cohort, three sources of information were available: (i) the reported administrative coverage – which we ignored because it was an implausible 120%; (ii) the results of the 2005–2006 National Family Health Survey – which we ignored, for all states, because of concerns over the collection of vaccination histories; and (iii) the results of a 2006 coverage evaluation survey, on which we based our estimate of coverage.

Our estimates of coverage were often only based on survey results, which frequently challenged the corresponding reported administrative coverages. For example, of the 253 estimates made of coverage for DTP3, 139 (55%) were based solely on survey results, 25 (10%) on reported administrative data and the remaining 89 (35%) on interpolation between – or extrapolation from – anchor points. Similar patterns were observed for the other vaccinations we investigated.

**Fig. 2:**
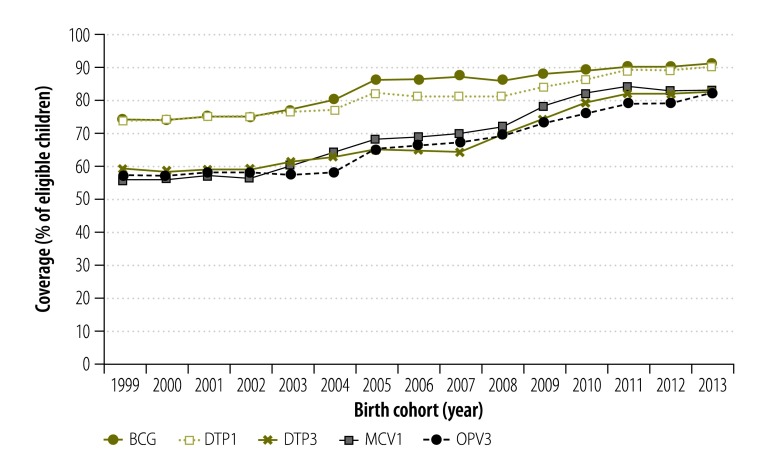
Estimates of national coverage with five routine child vaccinations, India, 1999–2013

BCG: bacille Calmette–Guérin vaccine; DTP1: first dose of vaccine against diphtheria, tetanus and pertussis; DTP3: third dose of vaccine against diphtheria, tetanus and pertussis; MV1: first dose of measles vaccine; OPV3: third dose of oral polio vaccine.

Where available, the results of nationally representative surveys were found to be generally supportive of our estimates of national coverages, which we derived from the state-level values (Fig. 2). Across vaccines, our estimates of national coverages tended to be lower than the corresponding reported administrative coverages (Fig. 2).

Between 1999 and 2013, estimated national coverages tended to gradually increase: from 74% to 91% for bacille Calmette–Guérin vaccine; from 74% to 90% and from 59% to 83% for DTP1 and DTP3, respectively; from 57% to 82% for the third dose of oral polio vaccine; and from 56% to 83% for the first dose of vaccine against measles. Most of these increases had occurred by 2010 and coverages seem to have plateaued between 2010 and 2013. Over the same period, there appeared to be substantial reductions in drop-out between DTP1 and DTP3.

In the sensitivity analysis, the assumption that coverage in the 18 unreviewed states and territories was not 100% but similar to that in the reviewed states changed our estimates of national coverages by less than two percentage points. Even when we assumed that coverage in each of the unreviewed states was only 62% – i.e. the lowest coverage estimated for any the reviewed states – the impact on the national estimates of coverage was 1.7%.

**Fig. 3:**
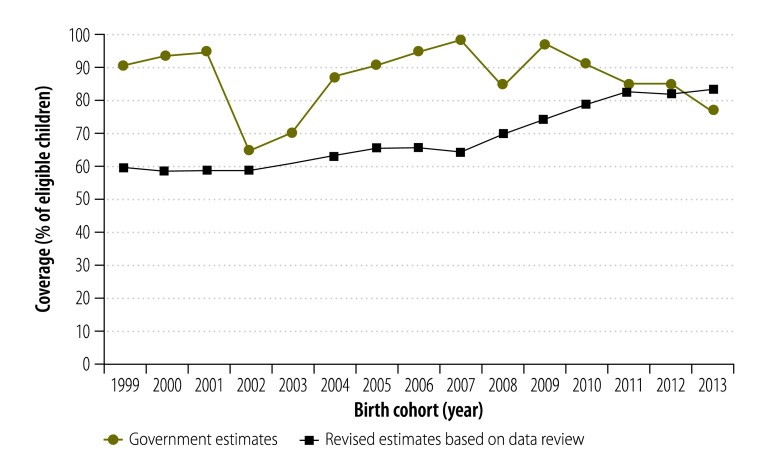
Estimates of national coverage for routine child immunization with the third dose of vaccine against diphtheria, tetanus and pertussis, India, 1999–2013

Previous estimates of national child vaccination coverage for India, over our study period, were largely based on reported administrative coverages (Fig. 3). These earlier estimates indicated large, rapid and implausible swings in coverage with DTP3 and surprisingly good levels of coverage given the programmatic activity and investment. In contrast, our estimates of national coverages indicate a consistent pattern of slow, steady and more plausible improvements in coverage (Fig. 3).

## Discussion

The results reported here reflect efforts by the Government of India to review the available data on state-level vaccination coverage systematically and produce better state-level and national estimates of coverage. They also reflect a growing awareness of the challenges of estimating actual vaccination coverages from reported administrative coverages of varying – and often unknown – quality. In July 2015, the Government of India used our results when it reported the performance of its national immunization system to WHO and UNICEF.^10^ We plan to continue our estimations at national level and extend the exercise to facilitate the estimation of district-level coverages within individual states.

Even after some correctable deficiencies in the coverage monitoring system were identified, improvements in the routine immunization of infants and children in India were held back by poor data on coverage. The desire to eliminate polio has driven some useful changes in India, including the use of microplanning, the development of the Reaching Every District strategy^11^ and the use of neonatal registration and tracking – primarily intended for activities against polio – to establish accurate child listings at local level. A revised strategy for the monitoring of routine immunizations was launched in July 2009. A transition in the reporting of administrative coverage, from a paper-based system to an electronic one, forms part of the development of a nationwide system of electronic data management that already includes the Health Management Information System and the Mother and Child Tracking System.^12^

While we recognize the critical importance of improving the quality of information on vaccination coverage from administrative reporting systems, we are also cognizant of the expected continued need for state-level surveys and improved rapid-monitoring exercises. Although the method of our data review may have fallen short of optimal, we found both the review and subsequent estimation exercise to be useful. The approach we followed allowed the evaluation and synthesis of multiple sources of coverage data, permitted some judgment of data quality and promoted a commitment to improved documentation.

Fig. 1 illustrates the considerable differences we observed between the reported administrative coverages and our corresponding estimates of actual coverage. Differences between information sources tended to grow smaller as time progressed – presumably as the newly introduced electronic reporting systems and initiatives to improve data quality began to mature.

Our approach to the estimation of coverages included the use of existing data, the assessment of all available state–vaccine–year combinations, the inclusion of diverse data sources, the application of a set of logical inference rules to the data review, use of input from stakeholders and the flexibility to override the inference rules – with justification and documentation of the decisions taken. This approach is only as good as the available data. Missing data and data of poor quality restrict attempts to produce accurate estimates. Our estimates of coverage remain subject to error and may well be inaccurate even when they appear to be well supported by data from multiple sources.^13^

The annual collection of data for the estimation of national vaccination coverages may well be critical to evaluating progress in the elimination of vaccine-preventable diseases. In India, although ever more children receive the benefits of vaccination, many children remain unvaccinated. Our estimates indicate that 80–85% of the Indian 2013 birth cohort received DTP3, third doses of oral polio vaccine and first doses of vaccine against measles. Although these results indicate that there have been substantial increases in coverage since 1999, much work will still be needed to reach Mission Indradhanush’s coverage goals. The government continues to address the challenges of documenting who is being missed by routine immunization services – and the reasons why they are being missed – through the design and implementation of innovative and targeted approaches to reach all children. At the same time, efforts are being implemented to ensure that appropriate commitment and investment are being made and that coordinated and coherent action is being taken to improve India’s programmes of routine immunization.
